# Predation, evo-devo, and historical contingency: A nematode predator drives evolution of aggregative multicellularity

**DOI:** 10.1101/2025.05.04.652091

**Published:** 2025-05-07

**Authors:** Kaitlin A. Schaal, Marco La Fortezza, Gregory J. Velicer

## Abstract

Research into the evolution of multicellularity often focuses on clonal multicellularity, yet aggregative multicellularity (AM) may respond to different drivers and is also highly interesting evolutionarily, for example in its behavioral, regulatory, morphological, and social complexity and diversity. We investigate the potential for predation to shape AM evolution across different combinations of three species comprising a multi-trophic food web. Together in a three-species community, the fruiting bacterium *Myxococcus xanthus* is a mesopredator, while the bacterivorous nematode *Pristionchus pacificus* is apex predator and the bacterium *Escherichia coli* is a shared basal prey for both predators. The number and morphology of *M. xanthus* fruiting bodies is found to respond evolutionarily to nematodes, regardless of whether *E. coli* is present. *E. coli* alone with *M. xanthus* tends to reduce both fruiting body formation and spore production, but adding nematodes eliminates those negative effects. *M. xanthus* lineages with an ancestral antibiotic-resistance mutation evolved less overall, revealing strong historical contingency and suggesting potential tradeoffs between antibiotic-resistance and responsiveness to biotic selection. Our results suggest that predation both of and by mesopredators has played important roles in the evolution of aggregative multicellularity and reveal complex inter-trophic evolutionary interactions in a relatively simple three-species food web.

## Full Text Availability

The license terms selected by the author(s) for this preprint version do not permit archiving in PMC. The full text is available from the preprint server.

